# Neuropsychiatric manifestations of Brain Sagging Syndrome. Case report and Literature review

**DOI:** 10.1192/j.eurpsy.2025.1694

**Published:** 2025-08-26

**Authors:** L. C. Ortega Sandoval, E. Lanchares Balboa, E. Abalde Garcia, J. I. Vizoso

**Affiliations:** 1 Hospital Alvaro Cunqueiro, Vigo, Spain

## Abstract

**Introduction:**

The presentation of psychotic symptoms in adults requires a global medical assessment, especially in cases of atypical presentations or if warning signs are present. The presence of cognitive symptoms and behavioral changes requires screening for various neurological diseases.

**Objectives:**

Underline the importance of neurological evaluation in atypical psychotic conditions with cognitive and behavioral symptoms. Describe Brain Sagging Dementia as a possible etiology of these conditions.

**Methods:**

Presentation of clinical case and bibliographic review.

**Results:**

The clinical case of a 59-year-old female patient brought to the emergency department for psychiatric evaluation due to behavioral alterations is described. During the evaluation, paranoid symptoms were detected, with marked suspicion towards her family, which led to her admission for psychiatric hospitalization. During observation, the clinical history was completed, revealing marked changes in behavior, apathy, perseveration, and decreased functionality for more than five years. Neuropsychological tests were performed, where cognitive and visuospatial alterations were evident. A consultation with the neurology service was requested, who initially considered the diagnosis of behavioral-variant frontotemporal dementia.

Given the history of orthostatic headaches secondary to cerebrospinal fluid hypotension due to a dorsal fistula, a new brain MRI was performed, which found evidence of cerebrospinal fluid hypotension without frontotemporal atrophy. Given all the clinical and radiological findings, a possible diagnosis of Brain Sagging Dementia was considered.

Brain Sagging Dementia is a rare syndrome caused by spontaneous intracranial hypotension (SIH), which mimics the behavioral clinical findings of frontotemporal dementia (bvFTD), excluding it due to the absence of frontotemporal atrophy. It is insidious in nature, with gradual cognitive and behavioral alterations.

The first-line treatment is an epidural blood patch, with partial resolution of symptoms in up to 81% of cases and complete resolution in up to 67%.

In this case presented, the patient is awaiting evaluation by neurosurgery.

**Conclusions:**

In case of suspected neurological origin of psychiatric symptoms, a complete evaluation is essential with special attention to potentially reversible causes.

It is important to keep in mind the neuropsychiatric manifestations that can occur in dementia and other neurology conditions, to avoid delaying a correct diagnosis. These include behavioral alterations, psychotic symptoms, eating disorders, as well as affective disorders ranging from apathy and depression to expansiveness with signs of disinhibition.

Brain sagging dementia is a reversible condition with symptoms of bvFTD, whose early diagnosis and treatment significantly improve the medical prognosis.

**Disclosure of Interest:**

None Declared

